# The capsule of *Porphyromonas gingivalis *reduces the immune response of human gingival fibroblasts

**DOI:** 10.1186/1471-2180-10-5

**Published:** 2010-01-11

**Authors:** Jorg Brunner, Nina Scheres, Nawal B El Idrissi, Dong M Deng, Marja L Laine, Arie J van Winkelhoff, Wim Crielaard

**Affiliations:** 1 Department of Oral Microbiology, Academic Centre for Dentistry Amsterdam, Universiteit van Amsterdam and Vrije Universiteit, Amsterdam, The Netherlands; 2 Center for Dentistry and Oral Hygiene, Department of Oral and Medical Microbiology, University Medical Center Groningen, Groningen, The Netherlands

## Abstract

**Background:**

Periodontitis is a bacterial infection of the periodontal tissues. The Gram-negative anaerobic bacterium *Porphyromonas gingivalis *is considered a major causative agent. One of the virulence factors of *P. gingivalis *is capsular polysaccharide (CPS). Non-encapsulated strains have been shown to be less virulent in mouse models than encapsulated strains.

**Results:**

To examine the role of the CPS in host-pathogen interactions we constructed an insertional isogenic *P. gingivalis *knockout in the epimerase-coding gene *epsC *that is located at the end of the CPS biosynthesis locus. This mutant was subsequently shown to be non-encapsulated. K1 capsule biosynthesis could be restored by *in trans *expression of an intact *epsC *gene. We used the *epsC *mutant, the W83 wild type strain and the complemented mutant to challenge human gingival fibroblasts to examine the immune response by quantification of *IL-1β*, *IL-6 *and *IL-8 *transcription levels. For each of the cytokines significantly higher expression levels were found when fibroblasts were challenged with the *epsC *mutant compared to those challenged with the W83 wild type, ranging from two times higher for IL-1β to five times higher for IL-8.

**Conclusions:**

These experiments provide the first evidence that *P. gingivalis *CPS acts as an interface between the pathogen and the host that may reduce the host's pro-inflammatory immune response. The higher virulence of encapsulated strains may be caused by this phenomenon which enables the bacteria to evade the immune system.

## Background

*Porphyromonas gingivalis *is a major pathogen in destructive periodontal diseases including chronic and aggressive periodontitis that are characterized by breakdown of the tooth-supporting tissues [1-3]. *P. gingivalis *is a black pigmented, often encapsulated, strict anaerobic, Gram negative coccobacillus that occurs in the human oral cavity.

Among the variety of virulence factors that have been described for *P. gingivalis*, CPS has shown to be a major factor in experimental infections. Studies in a mouse infection model have revealed that encapsulated *P. gingivalis *strains are more virulent than non-encapsulated strains [4-7]. Non-encapsulated strains mostly cause non-invasive, localized abscesses whereas encapsulated strains cause invasive, spreading phlegmonous infections after subcutaneous inoculation of experimental animals.

Six distinct capsular serotypes have currently been described (K1-K6) [8,9] and a seventh serotype (K7) has been suggested by R. E. Schifferle (personal communication). Small differences in virulence have been found between capsular serotypes and strong variation in virulence has been described between strains of the same capsular serotype [10]. CPS of all serotypes has been tested for induction of immunological responses in macrophages and it has been revealed that the CPS of K1 serotype strains induces higher chemokine expression in murine peritoneal macrophages than the other serotypes [11]. These data suggest that the K1 CPS plays an important role in host-pathogen interaction. The chemical composition of the K1 CPS has been studied to a limited extent. It has been reported that the CPS of K1 (strain W50) comprises of mannuronic acid (ManA), glucuronic acid (GlcA), galacturonic acid (GalA), galactose and N-acetylglucosamine (GlcNAc), but the CPS structure has not been solved [12].

Although CPS is a major structure at the interface between the bacterial cell and the host, the exact role of *P. gingivalis *CPS is not yet clear. Adhesion to epithelial cells has been shown to be higher for non-encapsulated *P. gingivalis *and the level and mechanism of co-aggregation has been shown to be CPS dependent [5,13,14]. In many pathogens CPS has been found to be involved in evasion of the host immune system by circumvention of phagocytosis, opsonization and complement killing [15-17].

The aim of this study was to investigate *in vitro *differences in host response during infection with a wild type and an isogenic non-encapsulated mutant of a naturally encapsulated strain. The well-studied K1 serotype W83 strain was used as the wild type strain since its CPS biosynthesis locus has been described [18,19]. An insertional mutation in *PG0120 *(*epsC*) was constructed, which yielded a non-encapsulated strain. The gene has been annotated as a UDP-GlcNAc 2-epimerase.

This *epsC *mutant is tested in a fibroblast infection model [20] since fibroblasts are the most abundant stromal cells in soft connective tissue of the gingiva [21] and among the first cells encountering periodontal infections by anaerobic bacteria like *P. gingivalis*. And above all, fibroblasts have been shown to be involved in the immune response in periodontitis [22,23]. Human gingival fibroblasts were infected with W83 and the *epsC *mutant and transcription of *IL-1β*, *IL-6 *and *IL-8 *was determined as host response parameters. This study provides the first direct evidence that *P. gingivalis *CPS reduces the host immune response, thereby potentially enabling evasion of the immune system to sustain successful long-term infection.

## Results

### *EpsC *mutant construction

**Figure 1:**
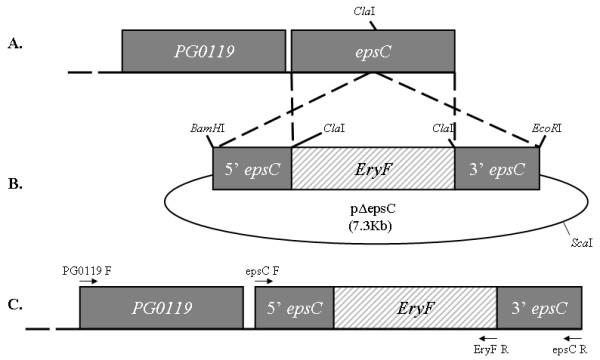
**Schematic representation of the knockout strategy to construct the *epsC *insertional mutation in W83**. A. The genetic arrangement of the 3'-end of the CPS locus in the W83 wild type strain with the grey rectangles representing the genes present. B. Construct pΔepsC for insertional inactivation of *epsC*. The 1.2 Kb *epsC *was inserted into *BamH*I-*EcoR*I digested *pGEX-6-p3 *(oval) and interrupted by insertion of a 1.2 Kb *EryF *(shaded rectangle) in the single *ClaI *restriction site present. The dashed lines between A and B show the homologous crossover regions between the plasmid and W83 CPS locus. C. The final arrangement of the 3'-end of the *P. gingivalis *CPS locus after double crossover showing the insertional inactivation of *epsC*. Arrows represent the primers used to confirm the integrity of the *epsC *mutant.

After transformation of the linearized plasmid pΔEpsC to *P. gingivalis *W83 the *epsC *insertional mutation was confirmed by specific PCR amplifications and agarose gel electrophoresis of the products (data not shown). Primer combinations epsC BamHI F × PG0119 R and EryF F × epsC EcoRI R (Table 1) ensured that a 1.2 Kb fragment of pΔEpsC had been integrated by double crossover at *PG0120 *(*epsC*) as expected, replacing the intact copy with the insertionally inactivated copy (Figure 1).

**Table 1:** Primers used in this study

Target	Name	Sequence (5'-3')
*epsC*	epsC BamHI F	ATATA**GGATCC**ATGAAAAAAGTGATGTTGGTC
	epsC EcoRI R	CTAT**GAATTC**ATCTTCGGCTAAATGCATCG
	epsC AscI F	GAATATA**GGCGCGCC**ATGAAAAAAGTGATGTTGGTC
	epsC SpeI	CTAT**ACTAGT**ATCTTCGGCTAAATGCATCG
*eryF*	eryF ClaI F	CCACC**ATCGAT**CGATAGCTTCCGCTATTGC
	eryF ClaI R	CCACC**ATCGAT**GTTTCCGCTCCATCGCCAATTTGC
*CP25*	CP25 ClaI F	GCCAT**ATCGAT**GCATGCGGATCCCATTATG
	CP25 AscI R	CCTTTA**GGCGCGCC**CTTAATTTCTCCTC
*IL-6*	IL-6 F	GGCACTGGCAGAAAACAACC
	IL-6 R	GGCAAGTCTCCTCATTGAATCC
*IL-8*	IL-8 F	GGCAGCCTTCCTGATTTCTG
	IL-8 R	CTGACACATCTAAGTTCTTCTTTAGCACTCCTT
*IL-1β*	IL-1β F	AAGATTCAGGTTTACTCACGTC
	IL-1β R	TGATGCTGCTTACATGTCTCG
*hup-1*	hup-1 F	GAAAAGGCCAACCTCACAAA
	hup-1 F	TCCGATGAGAGCGATTTTCT
*glk*	glk F	ATGAATCCGATCCGCCACCAC
	glk R	GCCTCCCATCCCAAAGCACT

In bold: restriction sites used in this study

To examine if the mutation had an influence on the growth characteristics of the *epsC *mutant both W83 and the *epsC *mutant were grown in brain heart infusion broth supplemented with hemin (5 μg/ml) and menadione (1 μg/ml) (BHI+H/M). Phase-contrast microscopy revealed that the mutant grows in aggregates, but no difference in growth rate was observed.

### *EpsC *mutant characterization

The potential polar effect of the insertional inactivation on the down stream gene of *epsC *named *hup-1 *was examined. Total RNA was extracted from W83 and the *epsC *mutant in the early exponential phase and the *hup-1 *expression levels were evaluated by Real-Time PCR. No significant difference in expression of *hup-1 *was found between W83 and the *epsC *mutant (data not shown).

To show the effect of capsule-loss on the surface structure of *P. gingivalis *the hydrophobicity of the *epsC *mutant was tested by the capacity to adhere to hexadecane. While 3% of W83 cells was shown to adhere to hexadecane more than 60% of the *epsC *mutant cells was adhered to hexadecane. 19% of the complemented mutant cells was adhered to hexadecane (see Additional file 1).

**Figure 2:**
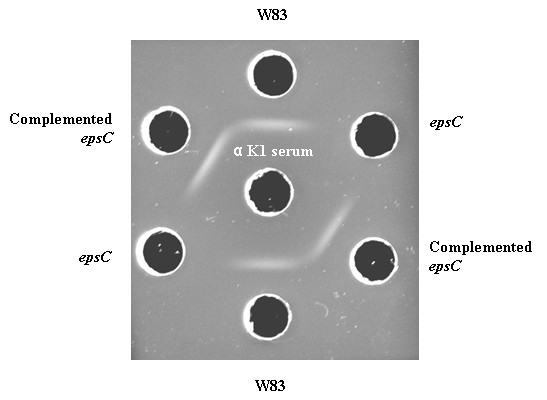
**Double immunodiffusion analysis of autoclaved supernatants of *P. gingivalis *strains**. Samples of W83, the *epsC *mutant and the complemented mutant were tested against the K1-specific antiserum (central well). Note that the white precipitate indicating recognition of CPS with the antiserum is absent in case of the *epsC *mutant, whereas the intact *epsC *copy restores the wild-type K1 antiserum recognition in the complemented mutant.

**Figure 3:**
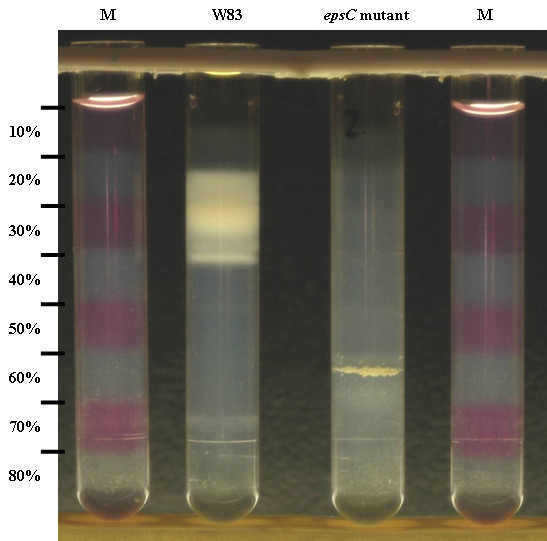
**Percoll density gradient centrifugation of W83 and epsC mutant**. 1 ml of a OD_690 _= 4 suspension of overnight-grown *P. gingivalis *was layered on top of a stepwise Percoll gradient (10-80%) and centrifuged at 8000 × g for one hour. The gradient is visualized using fuchsine-stained layers in the marker (M).W83 reproducibly settles in the interfaces of 10-20%, 20-30% and 30-40% where most of the bacterial material is found in the 20-30% interface. The *epsC *mutant settles as a distinct, granulous band at the 50-60% interface.

Reactivity with the CPS-specific polyclonal rabbit antisera against *P. gingivalis *serotypes K1-K6 [8,9] was examined for W83 and the *epsC *mutant. The *epsC *mutant was not recognized by any of the antisera including the K1 antiserum, whereas the wild type strain was only recognized by the K1 antiserum (Figure 2). Differences in CPS characteristics were also studied by Percoll density gradient centrifugation, which can reveal density differences between encapsulated and non-encapsulted *bacteroides *strains [24]. Percoll density gradient centrifugation analyses of W83 and the *epsC *mutant showed that the density of the mutant had been changed (Figure 3). Where W83 mostly settled at the 20-30% interface, the *epsC *mutant settled at the 50-60% interface. Note that the appearance of W83 is diffuse and not restricted to the 20-30% interface. The mutant settles as a compact and granulous layer.

**Figure 4:**
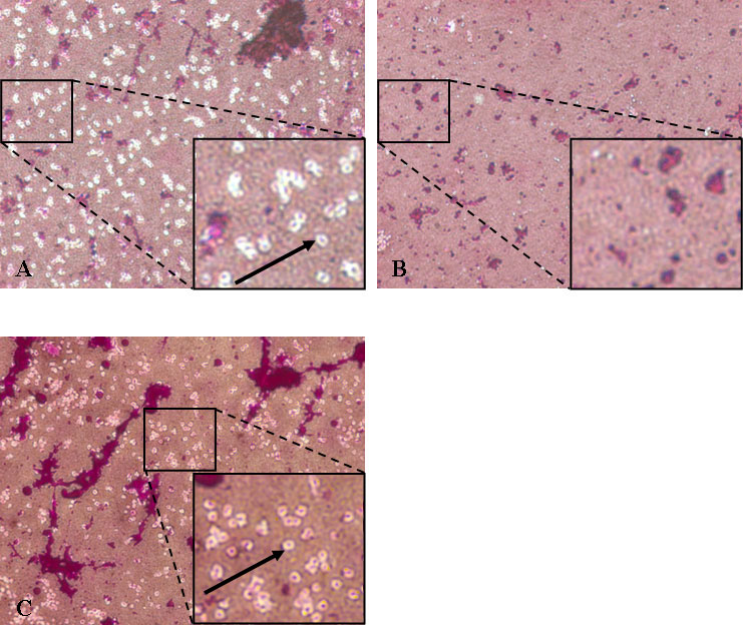
**Negative capsule staining of fuchsine-stained *P. gingivalis *cells with India Ink**. Phase contrast microscopic picture at a 1000× magnification of (A) W83 wild type strain, (B) *epsC *mutant and (C) the complemented *epsC *mutant in an India ink preparation which reveals the capsule as a white halo (arrow). The inset shows an extra six times magnification.

To conclusively examine the absence of CPS in the *epsC *mutant, light microscopy was performed using India ink in combination with fuchsine staining (Figure 4). The negative India ink staining allows direct visualization of the capsule, appearing as a light halo surrounding the *P. gingivalis *cell. Fuchsine is used to stain the cell body. The halos around the W83 wild type strain are clearly visible in the phase contrast microscopic picture, whereas halos are absent around the *epsC *mutant. The intact *epsC *gene *in trans *under control of the CP25 promoter rescues the wild-type phenotype enabling the complemented mutant to produce a K1 capsule again (Figures 2 and 4).

### Fibroblast response to *P. gingivalis *challenge

**Figure 5:**
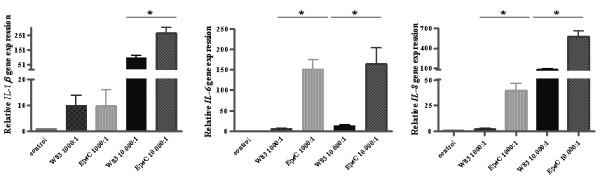
**Relative expression of *IL-1β*, *IL-6 *and *IL-8 *genes in human gingival fibroblasts (HGF1) infected with *P. gingivalis *W83 and the *epsC *mutant**. After a 6-hour challenge with *P. gingivalis *cells at MOI 1000:1 or 10.000:1 as indicated on the Y-axis, the expression levels of *IL-1β*, *IL-6 *and *IL-8 *in human gingival fibroblasts were measured using RT-PCR and represented as a relative value compared to a non-infected control sample which is set to a value of 1. Significant differences p < 0.01 are indicated by an asterisk.

To study the effect of the *epsC *deletion on the host immune response six hour infection studies of human gingival fibroblasts with W83 and the *epsC *mutant were performed. Figure 5 shows *IL-1β*, *IL-6 *and *IL-8 *expression of infected gingival fibroblasts relative to the non-infected negative control which is set to 1 and normalized against expression of housekeeping gene *GAPDH*.

At multiplicity of infection (MOI) 1000:1 of both strains a small induction of the tested genes could be detected compared to the non-infected control, but significant induction for all three genes was found when MOI 10.000:1 was used for infection.At MOI 1000:1 *IL-6 *and *IL-8 *expression showed a significantly higher induction (150-fold and 37-fold induction respectively) in the cells challenged with the *epsC *mutant when compared to the wild-type (6-fold and 2-fold induction respectively), *IL-1β *did not show a difference compared to the wild-type. However, when gingival fibroblasts were challenged with MOI 10.000 bacteria all three tested genes showed a significantly higher induction in the cells challenged with the *epsC *mutant than with W83 (figure 5). When fibroblasts were challenged with the complemented mutant the response was almost completely restored to wild-type levels (see Additional file 2).

Sedimentation of the *epsC *mutant in comparison to W83 was analyzed in the same buffer as used in the infection experiments. No significant sedimentation differences were found between W83 and the *epsC *mutant within the 6 hours needed for infection of the fibroblasts (data not shown).

Since infections were done with viable *P. gingivalis*, survival of the bacteria during the 6-hour aerobic period of infection in DMEM medium had to be ensured. Therefore a 6-hour survival experiment was performed in the 24-well plates used for the fibroblast challenge. On average 60-75% of W83, *epsC *mutant and complemented mutant cells survived for 6 hours in Dulbecco's modified Eagle's Medium (DMEM; Sigma Chemical Co.) supplemented with 10% fetal calf serum (FCS) (see Additional file 3).

## Discussion

The aim of this paper was to understand the role of *P. gingivalis *CPS in the response of human gingival fibroblasts.*P. gingivalis *CPS has been regarded as an important virulence factor. It has been shown to induce inflammatory mediators in *in vitro *studies [11]. The capsule also plays an important role in shielding of immune response inducers in several bacterial species [25-27]. Since a distinct CPS biosynthesis locus in *P. gingivalis *has been described and shown to be functional [18,19], studying the role of *P. gingivalis *CPS in the immune response by use of a mutant became feasible. For this purpose an insertional isogenic knockout in *epsC*, a potential capsular biosynthesis gene within the CPS biosynthesis locus present in strains of different serotypes, was constructed to prevent capsule synthesis. The homologue of this gene in *Listeria monocytogenes lmo2537 *has been shown to be essential for survival, and has been suggested to be involved in the maintenance of cell shape by providing a precursor of the teichoic acid linkage unit that serves as an acceptor for the main teichoic acid chain assembly [28]. Construction of the *P. gingivalis epsC *mutant shows that the *epsC *gene is not essential for *P. gingivalis *viability.

In the present study the mutant is shown to be non-encapsulated by double immuno-diffusion, density gradient centrifugation and India ink staining. Complementation resulted in rescue of wild-type K1 capsule biosynthesis. Although the exact role of *epsC *remains to be elucidated, this finding provides evidence that EpsC is essential in *P. gingivalis *CPS biosynthesis.

The *epsC *mutant was expected to have altered immunological properties. To examine the role of CPS, both the wild-type and the *epsC *mutant were used in an *in vitro *challenge of primary human gingival fibroblasts. Since the *epsC *mutant has altered physical properties, it was important to compare the sedimentation rate and viability of both the wild type and the mutant strain since these could have influenced the amount of living bacterial cells that are in contact with the fibroblasts. No differences were observed between the strains during the 6 hours of infection.

From the infection experiments of the gingival fibroblasts it became apparent that pro-inflammatory mediators *IL-1β*, *IL-6 *and *IL-8 *expression levels were up-regulated after a 6-hour challenge with both wild-type W83 and the *epsC *mutant in comparison to the non-infected control, especially when MOIs of 10.000:1 were used.

A challenge with the *epsC *mutant induced a significantly higher pro-inflammatory immune response than a challenge with the wild type W83, as shown by *IL-1β*, *IL-6 *and *IL-8 *gene expression. So, even though purified *P. gingivalis *CPS has been shown to stimulate pro-inflammatory cytokine expression in murine peritoneal macrophages [11] the absence of capsule induces extra cytokine induction when viable *P. gingivalis *cells were used to challenge fibroblasts.

Capsular polysaccharides of several bacteria have been implicated in down-regulation of pro-inflammatory cytokine production, including *Klebsiella pneumonia *[29]. *Bacteroides fragilis *capsular polysaccharide complex has been shown to induce IL-10 expression, a regulating cytokine which may cause suppression of the immune system [30].

An explanation of our results may be that the CPS prevents more potent immune inducers to be recognized by Toll-like receptors on the fibroblasts. It has been shown that the capsular antigen in *Salmonella typhi*, referred to as Vi-antigen, is able to prevent Toll-like receptor 4 recognition of LPS, thereby reducing expression of pro-inflammatory TNF-α and IL-6 [31-33]. In *E. coli *the capsule may cover short (10 nm) bacterial adhesins, which do not penetrate the 0.2-1.0 μm capsular layer, preventing them from being recognized by the immune system [26]. Likewise, *P. gingivalis *strain W83 was described as to have a small amount of short fimbriae that might be mostly covered by the CPS [34].

Another or additional explanation of our findings could be immune suppression by *P. gingivalis *CPS, meaning that CPS would actively modulate the immune response of the fibroblasts, leading to lower inflammatory cytokine expression levels, potentially enabling *P. gingivalis *to evade the immune system.

For several bacteria it has been described that capsular biosynthesis can be modulated depending on environmental conditions [35,36]. Although presently no regulation of *P. gingivalis *capsule expression has been described, we can not exclude the possibility that in the *in vivo *situation capsule expression is regulated. However, the reduced pro-inflammatory host's immune response by the encapsulated strain may explain the documented differences between natural *P. gingivalis *strains in spreading. Whereas non-encapsulated strains are tackled directly by the immune system in localized abscesses, the more virulent encapsulated strains can evade this defence and cause phlegmonous infections [4-7].

## Conclusions

The epimerase-coding gene *epsC *of *P. gingivalis *is essential for CPS synthesis. The absence of CPS results in increased induction of *IL-1β*, *IL-6 *and *IL-8 *in human gingival fibroblasts upon *in vitro *infection with viable *P. gingivalis *cells. *P. gingivalis *CPS acts as a functional interface between the pathogen and the host. The CPS-related reduced pro-inflammatory response can explain why natural non-encapsulated strains cause localized abscesses and encapsulated strains spreading phlegmonous infections.

## Methods

### Bacterial maintenance

*P. gingivalis *strains were grown either on 5% horse blood agar plates (Oxoid no. 2, Basingstoke, UK) supplemented with hemin (5 μg/ml) and menadione (1 μg/ml) (BA+H/M plates) or BHI+H/M, both, at 37°C in an anaerobic atmosphere of 80% N_2_, 10% H_2_, and 10% CO_2_. Mutants were selected in the presence of 5 μg/ml erythromycin. Complemented mutants were selected in the presence of 50 μg/ml gentamycin and 1 μg/ml tetracycline. Purity of *P. gingivalis *liquid and plate-grown cultures was routinely checked by gram staining and microscopic examination.

*Escherichia coli *DH5α was used for maintenance and construction of plasmids. DH5α was cultured in Luria-Bertani (LB) broth or on solid medium (LB broth with addition of 1.5% agar). Ampicillin (Na^+ ^salt; 100 μg/ml) was added to the growth media to select for pUC-derived plasmids. *E. coli *S17-1 grown on LB supplemented with 5 μg/ml tetracycline carrying the complementation construct pT-PG0120 was used for conjugation with *P. gingivalis*.

### Human gingival fibroblasts

The gingival fibroblasts (HGF1 and HGF2) used in this study were collected from extracted third molars of two periodontally healthy subjects with a high pro-inflammatory immunological response when challenged with *P. gingivalis *[20]. Donors had given written informed consent, and the study was approved by the VUmc Medical Ethical committee.

### Genomic DNA isolation from *P. gingivalis*

Genomic DNA from *P. gingivalis *strains was isolated from plate-grown bacteria using the DNeasy tissue kit (Qiagen Benelux BV). The DNA concentration of all samples after purification was between 20 ng/μl and 60 ng/μl.

### Generation of an insertional knockout construct for *epsC*

To make an insertional knockout of *epsC *in the W83 wild type strain we constructed plasmid pΔEpsC. Primers epsC BamHI-F and epsC EcoRI-R (see table 1 for details) were used to amplify the 1.2 Kb *epsC *gene from *P. gingivalis *W83 genomic DNA in a PCR reaction. *Pfu *polymerase (Fermentas, GmbH, St. Leon-Rot, Germany) was used according to the manufacturer's protocol with 100 ng of genomic DNA. The PCR program started with 95°C for 5 min and then 25 cycles of 95°C, 55°C and 72°C for 30 s, 30 s and 2.5 min respectively and was ended by one step of 72°C for 5 min. The amplified fragment was cleaned using the Qiagen PCR purification kit (Qiagen Benelux B.V.) and restricted with *Bam*HI and *Eco*RI. This restricted *epsC *gene fragment was ligated into *Bam*HI-*Eco*RI restricted pGEX-6p-3 plasmid to yield pGEX-PG0120.

The 1.2 Kb EryF erythromycin resistance cassettes for use in *P. gingivalis *was amplified from plasmid pEP4351 using primers EryF ClaI F and EryF ClaI R. and after restriction with *Cla*I this fragment was ligated into the *Cla*I-restricted pGEX-PG0120 plasmid yielding pΔEpsC. The *Sca*I-linearized pΔEpsC plasmid was used for insertional inactivation of *epsC *in *P. gingivalis *strain W83.

### Complementation of the *epsC *mutant

The 120 bp artificial constitutive CP25 promoter [37] was amplified from plasmid pDM15 [38] using primers CP25 ClaI F and CP25 AscI R. The intact *epsC *1.2 Kb gene was amplified from genomic DNA of *P. gingivalis *strain W83 using primers epsC AscI F and epsC SpeI R. After ligation of these fragments into cloning vector pJET1.2 (Fermentas, GmbH, St. Leon-Rot, Germany) the constructed expression cassette was cut out with *Xho*I and *Hind*III and ligated into the *Sal*I and *Hind*III digested pT-COW shuttle plasmid [39] to yield the complementation construct pT-PG0120.

### Transformation of *P. gingivalis*

BHI+H/M was inoculated with *P. gingivalis *W83 from a 6-day-old blood agar plate. This pre-culture was anaerobically incubated at 37°C for 2 days. 2 ml of the pre-culture was used to inoculate a 100 ml culture. The next day this culture was used to inoculate 2 × 100 ml of fresh BHI+H/M to an OD_690 _of 0.2. After six hours of anaerobic incubation at 37°C the cells were harvested by centrifugation in mid-exponential phase. The pellet was washed two times in 20 ml EPB (10% glycerol, 1 mM MgCl_2_) and after that resuspended in 2 ml of EPB. Aliquots of 200 μl were stored at -80°C and used for electroporation.

200 ng of *Pst*I digested pΔEpsC was added to 200 μl of W83 *P. gingivalis *cells. The mixture was transferred to a 2 mm electroporation cuvette and electroporated using an Electro Cell Manipulator 600 (BTX Instrument Division, Holliston, MA, USA; 25 μF, 2.5 kV, 186 Ω). 1 ml of BHI+H/M was added immediately after the pulse. The cells were left for recovery anaerobically at 37°C for 18 hours. The suspension was plated on BA+H/M plates with 5 μg/ml erythromycin for selection of the transformants. The authenticity of the insertional knockout *epsC *mutants was verified using primer combinations epsC BamHI F × PG0119 R and EryF ClaI F × epsC EcoRI R. Furthermore, using Real-Time PCR, the expression of the downstream gene *hup-1 *in both W83 and the *epsC *mutant was monitored using primers hup-1 F and hup-1 R to exclude polar effects. W83 and the *epsC *mutant were grown till early exponential phase. The cell pellets were collected by centrifugation and resuspended in RLT buffer (Qiagen, Benelux B. V.). The cells were disrupted using a Fast Prep Cell Disrupter (Bio 101, Thermo electron corporation, Milford, USA) and centrifuged, the total RNA was extracted from the supernatant according to the manufacturer's protocol of Qiagen RNeasy^® ^mini kit (Qiagen Benelux B.V.). The residual contaminating genomic DNA was removed by Turbo DNA-free™ kit (Ambion, Austin, USA). mRNA was then reverse transcribed using the Fermentas first-strand cDNA synthesis kit (Fermentas GmbH, St. Leon-Rot, Germany) according to the manufacturer's protocol.

The synthesized cDNA was further analyzed using Real-Time PCR with gene-specific primers on an ABI Prism 7000 Sequence Detecting System (Applied Biosystems, Nieuwerkerk a/d lJssel, The Netherlands). Gene expression was normalized to the expression of glucokinase (*glk*), amplified with primers glk F and glk R [40]. The relative *hup-1 *expression levels of W83 from three independent experiments were compared in duplicate to those of the *epsC *mutant.

### Conjugation of *P. gingivalis*

To complement the *epsC *mutant, plasmid pT-PG0120 was transferred into the mutant by conjugation following a protocol described earlier [41], with slight modifications. For selection of *P. gingivalis *after the over-night conjugation we used 50 μg/ml of gentamycin in our blood agar plates instead of 150 μg/ml. Integrity of the trans-conjugants was confirmed by colony PCR and plasmid isolation combined with restriction analysis using a plasmid isolation kit (Qiagen Benelux B.V.).

### Percoll density gradient centrifugation

Percoll density gradients were in principle prepared as described by Patrick and Reid [24]. In short, a 9:1 stock solution of Percoll (Pharmacia, Biotech AB, Uppsala, Sweden) was prepared with 1.5 M NaCl. Solutions containing 80, 70, 60, 50, 40, 30, 20 and 10% Percoll in 0.15 NaCl were prepared from the stock. In an open top 14 ml polycarbonate tube (Kontron instruments, Milan, Italy) 1.5 ml of each of the solutions was carefully layered on top of the previous starting with 80%. 1 ml of an anaerobically grown over night culture of wild type and the *epsC *mutant concentrated to an OD_690 _of 4 in PBS was added to the top of the 10% layer and centrifuged for one hour at 8000 × g at 20°C in a Centrikon TST 41.14 rotor (Kontron instruments, Milan, Italy) using a Centrikon T-1170 (Kontron instruments, Milan, Italy) centrifuge.

### Hydrophobicty of *P. gingivalis*

W83, the *epsC *mutant and the complemented mutant were grown 18 hours in BHI+H/M. The bacteria were washed twice in PBS after which the OD_600 _was set to 0.5. After addition of 150 μl n-hexadecane to 3 ml of this suspension the mix was vortexed 30 seconds, rested for 5 seconds and vortexed for 25 seconds. After exactly 10 minutes incubation at room temperature a sample was taken to measure the OD_600 _of the aqueous phase. The percentage of bacteria adhered to hexadecane was calculated by the formula: (OD_600 _before-OD_600 _after)/OD_600 _before × 100%. The presented data in Additional figure 1 were collected from two experiments using triplicate measurements.

### *P. gingivalis *serotyping

Serotyping of *P. gingivalis *was based on the detection of the six described K-antigens [8,9]. In short, serotype-specific, polyclonal antisera were obtained after immunization of rabbits with whole bacterial cells of the six *P. gingivalis *type strains [42]. Bacterial antigens for double immunodiffusion tests were prepared as described previously [8]. Immunodiffusion was carried out in 1% agarose (Sigma Chemical Co., St. Louis, MO, type 1, low EEO) in 50 mM Tris-HCl buffer (pH 8.6). 10 μl antiserum and 10 μl of antigen were loaded and allowed to diffuse and precipitate for 48 hours at room temperature.

### India ink negative staining

*P. gingivalis *cells were taken from 4 day-old plates and resuspended in 1 ml of PBS. On a glass slide 10 μl of this suspension was mixed with 10 μl of India ink (Talens, Apeldoorn, The Netherlands) and using another glass slide a thin film was made. The film was air-dried. A drop of 0.2% fuchsine was carefully added onto the film and removed after 2 minutes by decanting. Then the film was air-dried. Pictures were taken with a Leica DC500 camera on a Zeiss Axioskop using phase-contrast.

### Growth curve

Pre-cultures of W83 and the *epsC *mutant were grown anaerobically for 18 hours in BHI+H/M at 37°C. The pre-cultures were diluted to an OD_690 _of 0.05 *in duplo *in fresh BHI+H/M and incubated anaerobically at 37°C. Every few hours the OD_690 _was measured and a sample was taken for cfu-counts.

### Sedimentation of *P. gingivalis*

W83 and the *epsC *mutant were grown anaerobically for 18 hours in BHI+H/M at 37°C. After 3 wash steps in phosphate buffered saline (PBS) the OD_690 _was standardized to 5 in DMEM with 10% FCS. 10 ml of this culture was added to 40 ml DMEM with 10% FCS in a 100 ml flask to set the OD_690 _to 1. The cultures were incubated standing still at 37°C for six hours. At regular time intervals, a 200 μl sample was taken 0.5 cm from the liquid surface and the decrease of the OD_690 _values was determined as a measure for sedimentation.

### Survival of *P. gingivalis*

W83, the *epsC *mutant and the complemented mutant were grown anaerobically for 18 hours in BHI+H/M at 37°C. After 2 wash steps in phosphate buffered saline (PBS) the pellets were resuspended in DMEM with 10% FCS to an OD_690 _of 0.05 as used in fibroblast infections at MOI 10.000:1. 500 μl of these suspensions was incubated at 37°C in a humidified atmosphere of 5% CO_2 _in air. Samples for cfu-counts were taken at t = 0 hours, t = 3 hours and t = 6 hours and dilutions were plated on BA+H/M plates.

### Infection of gingival fibroblasts with *P. gingivalis*

Bacteria were grown overnight for 18 hours in BHI+H/M. The bacterial cells were washed three times in PBS and then used to infect gingival fibroblasts at MOIs of 1000:1 and 10.000:1 (bacteria cells: fibroblasts) in a total volume of 500 μl DMEM with 10% FCS in 24-well plates. The plates were incubated for 6 hours at 37°C in a humidified atmosphere of 5% CO_2 _in air. The cells were washed twice with cold PBS. Then 350 μl lysis buffer (1% β-mercapthanol in RLT buffer) was added to the cells according to the protocol of Qiagen RNeasy^® ^mini kit (Qiagen Benelux B.V.) after which the plate was stored at -80°C for later use.

### RNA isolation and reverse transcription

mRNA was isolated from the gingival fibroblast lysates according to the manufacturer's protocol of Qiagen RNeasy^® ^mini kit (Qiagen Benelux B.V.). The mRNA concentrations of the samples were determined using the Nanodrop ND_1000 (Isogen Life Science). mRNA was reverse transcribed using the Fermentas first-strand cDNA synthesis kit (Fermentas GmbH, St. Leon-Rot, Germany) according to the manufacturer's protocol.

### Real-Time PCR

cDNA synthesized from mRNA isolated from gingival fibroblasts after infection with *P. gingivalis *was analyzed in quadruple using Real-Time PCR with gene-specific primers on a ABI Prism 7000 Sequence Detecting System (Applied Biosystems, Nieuwerkerk a/d lJssel, The Netherlands). Reactions were performed with 2 ng cDNA in a total volume of 8 μl containing SYBR Green PCR Master Mix (Applied Biosystems) and 0.99 pM of each primer. After activation of the AmpliTaq Gold DNA polymerase for 10 minutes at 94°C, 40 cycles were run of a two step PCR consisting of a denaturation step at 95°C for 30 seconds and annealing and extension step at 60°C for 1 minute. Predicted product sizes were in the 100-200 bp range. Subsequently the PCR products were subjected to melting curve analysis to test if any unspecific PCR products were generated. The PCR reactions of the different amplicons had equal efficiencies. Samples were normalized for the expression of housekeeping gene *GAPDH*, which is not affected by the experimental conditions, by calculating the Δ Ct (Ct _housekeeping gene _- Ct _gene of interest_) and expression of the different genes is expressed as 2^-(ΔCt)^. Fold increase in gene expression (induction) was expressed by 2 ^-(ΔΔCt)^, wherein ΔΔCt = ΔCt_challenged_- average Ct-value _non-challenged_.

### Statistical analysis

Differences in gene induction between multiple groups were tested by one-way analysis of variance (ANOVA) and Bonferroni's Multiple Comparison Test. Tests were performed with GraphPad Prism version 4.00 for Windows, GraphPad Software, San Diego California USA. Differences were considered significant at p < 0.01.

## Authors' contributions

JB performed the cloning work, mutant construction, hydrophobicity test, density gradient centrifugation, negative staining, serotyping and drafted the manuscript. NBEI made the growth curves and did the sedimentation assay. NS and NBEI together performed the fibroblast infection experiments, the transcription analyses and statistical analyses. DMD analyzed the strains using Real-Time PCR and performed part of the statistical analysis. ML, AJvW and WC were involved in the study design, supervision and helped to draft the manuscript. All authors read and approved the final manuscript.

## Supplementary Material

Additional file 1 **Hydrophobicity of P. gingivalis strains**. Percentage of bacterial cells adhered to hexadecane after extensive vortexing and 10 minutes incubation. 3.4%, 61% and 19% of the cells was adhered to hexadecane for W83, the epsC mutant and the complemented mutant respectively, indicating increased hydrophobicity for the epsC mutant. The data are the averages of two experiments comprised of triplicate measurements. The bars show the standard deviations. Click here for file

Additional file 2 **Effect of complementation of the *epsC *mutant on the immune response mutant of human gingival fibroblasts (HGF2)**. After a 6-hour challenge with *P. gingivalis *cells at MOI 10.000:1, the expression levels of *IL-1β*, *IL-6 *and *IL-8 *in human gingival fibroblasts were measured using RT-PCR and if possible represented as a relative value compared to a non-infected control sample which is set to a value of 1. Relative *IL-1β *expression could not be calculated as *IL-1β *was not detected in the non-infected control. Complementation almost restored the wild-type situation for *IL-1β *(83%), *IL-6 *(83%) and *IL-8 *(77%). Click here for file

Additional file 3 **Six hour survival of W83, the *epsC *mutant and the complemented mutant under aerobic experimental conditions**. Survival of W83, the *epsC *mutant and the complemented mutant in 0.5 ml DMEM + 10% FCS under humidified 5% CO2 conditions was determined by cfu-counts on BA + H/M plates. Survival of 67%, 60 and 73% was found for each strain respectively. Error bars represent the standard deviations of triplicate measurements. Click here for file
