# Supplementary figures and images for: The capsule of Porphyromonas gingivalis reduces the immune response of human gingival fibroblasts
Source: BMC Microbiol. 2010 Jan 11;10:5. doi: 10.1186/1471-2180-10-5 (PMC2817674; doi:10.1186/1471-2180-10-5)

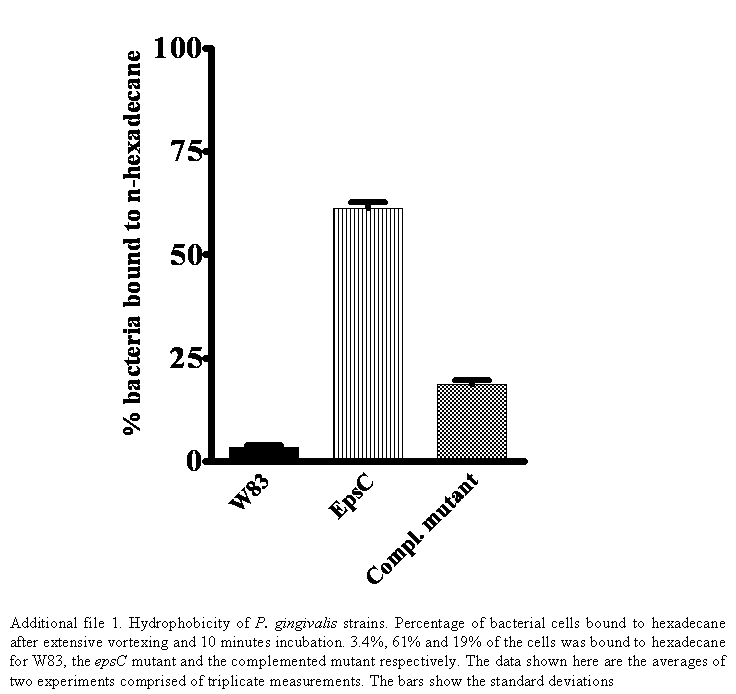

Supplement: Additional file 1 — Hydrophobicity of P. gingivalis strains. Percentage of bacterial cells adhered to hexadecane after extensive vortexing and 10 minutes incubation. 3.4%, 61% and 19% of the cells was adhered to hexadecane for W83, the epsC mutant and the complemented mutant respectively, indicating increased hydrophobicity for the epsC mutant. The data are the averages of two experiments comprised of triplicate measurements. The bars show the standard deviations. [file 1471-2180-10-5-S1.PNG]

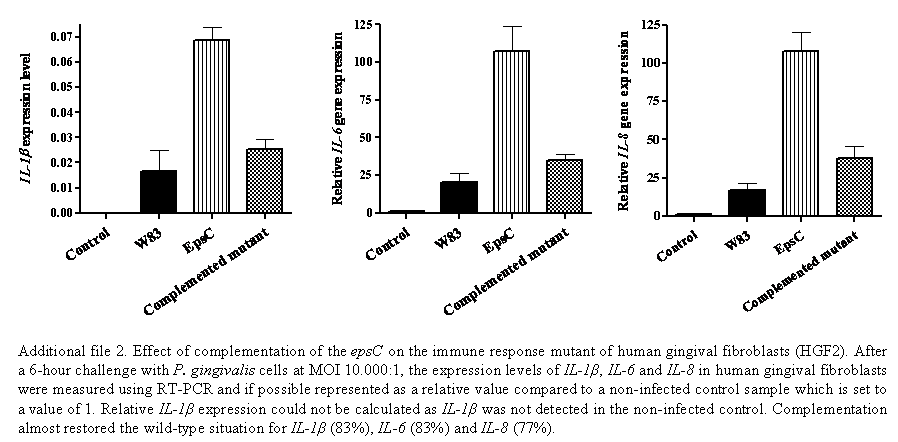

Supplement: Additional file 2 — Effect of complementation of the epsC mutant on the immune response mutant of human gingival fibroblasts (HGF2). After a 6-hour challenge with P. gingivalis cells at MOI 10.000:1, the expression levels of IL-1β, IL-6 and IL-8 in human gingival fibroblasts were measured using RT-PCR and if possible represented as a relative value compared to a non-infected control sample which is set to a value of 1. Relative IL-1β expression could not be calculated as IL-1β was not detected in the non-infected control. Complementation almost restored the wild-type situation for IL-1β (83%), IL-6 (83%) and IL-8 (77%). [file 1471-2180-10-5-S2.PNG]

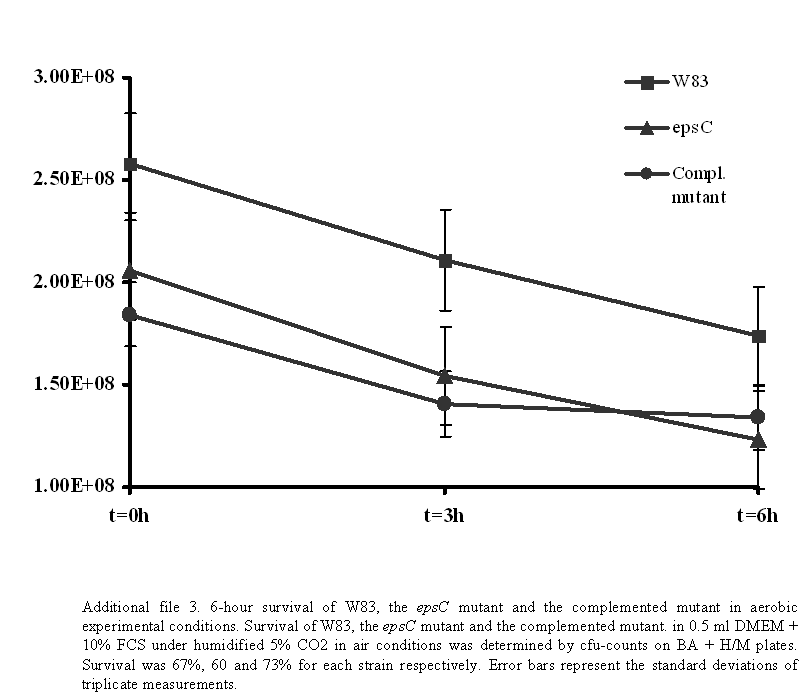

Supplement: Additional file 3 — Six hour survival of W83, the epsC mutant and the complemented mutant under aerobic experimental conditions. Survival of W83, the epsC mutant and the complemented mutant in 0.5 ml DMEM + 10% FCS under humidified 5% CO2 conditions was determined by cfu-counts on BA + H/M plates. Survival of 67%, 60 and 73% was found for each strain respectively. Error bars represent the standard deviations of triplicate measurements. [file 1471-2180-10-5-S3.PNG]
